# FRAILTY IN HEALTHY OLDEST OLD: CHARACTERIZING THE FRAILTY INDEX OF SUPER-SENIORS

**DOI:** 10.1093/geroni/igz038.3330

**Published:** 2019-11-08

**Authors:** Betty Chinda, Xiaowei Song*, Shirromi Sarveswaran, Kenneth Rockwood, Angela Brooks-Wilson*

**Affiliations:** 1 Department of Biomedical Physiology and Kinesiology, Simon Fraser University, Burnaby, British Columbia, Canada; 2 Health Research and Innovation, Surrey Memorial Hospital, Fraser Health Authority, Surrey, British Columbia, Canada; 3 Dalhousie University, Halifax, Nova Scotia, Canada; 4 Genome Sciences Centre, BC Cancer, Vancouver, British Columbia, Canada

## Abstract

People at advanced ages often have multiple comorbidities and high frailty. We characterized frailty in “Super-Seniors”, individuals 85 or older who have never been diagnosed with cancer, cardiovascular or lung disease, diabetes or dementia. Super-Seniors were enrolled in the Vancouver Healthy Aging Study that consisted of Phase1 (2004-2007; n=486; age=88.6±3.1 years; female=67.5%) and Phase2 (2014-2019; n=167; age=89.2±3.8 years; female=65.3%). A frailty index (FI) that assesses the accumulation of health deficits was calculated as the proportion of deficits present over those considered (here, 30). The FI distribution patterns, mean, median, 99% limit values, relationship to age, and sex differences were analyzed. The FI of Super-Seniors is right-skewed, with a mean of 0.19±0.09 (median=0.17; limit=0.54) in Phase1 and 0.22±0.08 (median=0.21; limit=0.47) in Phase2. Most Super-Seniors (79% and 61% in Phases 1 and 2) had ≤8 of the 30 deficits; FI≤0.24. The FI increased with age (r’s=0.29 and 0.24); women showed a higher mean FI than men. Data demonstrated the known and consistent characteristics of the FI. The Super-Seniors, who are healthier than the general population of oldest old, have a significantly lower FI that is more typical of individuals aged about 65. The low FI of these healthy oldest old is consistent with their health and high physical and cognitive function, and underscores their suitability for study as a healthy aged group. Further research will investigate how the FI of Super-Seniors is related to lifestyle and genetic factors and health outcomes.

